# The accuracy of different macrogeometry of dental implant in dynamic navigation guided immediate implant placement in the maxillary aesthetic zone: an in vitro study

**DOI:** 10.1186/s40729-025-00597-8

**Published:** 2025-03-26

**Authors:** Jinyan Chen, Xinbo Yu, Yiqun Wu, Feng Wang

**Affiliations:** 1 Division of Oral and Maxillofacial Surgery, Faculty of Dentistry, The University of Hong Kong, 2B95, 2/F, OMFS, the Prince Philip Dental Hospital, 34 Hospital Rd, Sai Ying Pun, Hong Kong, China; 2 Second Dental Center, Shanghai Ninth People’s Hospital, Shanghai Jiao Tong University School of Medicine; College of Stomatology, National Center for Stomatology; National Clinical Research Center for Oral Diseases; Shanghai Key Laboratory of Stomatology; Shanghai Research Institute of Stomatology, Shanghai Jiao Tong University, No. 280, Mohe Road, Baoshan District, Shanghai, China

**Keywords:** Implantology, Guided implant surgery, Navigation, Immediate implant placement, Macrogeometry

## Abstract

**Purpose:**

This study aimed to compare the accuracy of immediate implant placement (IIP) with different implant macrogeometry using a dynamic navigation in the maxillary aesthetic zone.

**Methods:**

Seventy-six extraction sockets in the maxillary aesthetic zone from nineteen partially edentulous models were randomly divided into four implant system groups with different macrogeometry: non-progressive and trapezoidal (NP-T), progressive and trapezoidal (P-T), progressive and V-shaped (P-V), progressive and spiral (P-S). The coronal, apical, and angular deviations of the fully guided implants with navigation were measured and compared among different groups.

**Results:**

Significant differences were detected in coronal, coronal buccolingual, coronal depth, apical, apical buccolingual, and apical depth deviations among the four groups (*p* = 0.035, *p* = 0.001, *p* = 0.004, *p* = 0.047, *p* = 0.007, *p* = 0.004, respectively). The P-V group demonstrated minimal coronal and apical buccolingual deviations (mean ± SD: 0.06 ± 0.35 mm and 0.00 ± 0.42 mm, respectively) for IIP with the guidance of dynamic navigation.

**Conclusions:**

With the limitation of the in vitro study, different microgeometry of implants might influence the accuracy of IIP in the maxillary aesthetic zone with dynamic navigation. Implants with progressive and V-shaped thread designs perform best in reducing buccolingual deviations.

## Background

Immediate implant placement (IIP) is often considered as an appealing treatment concept in the esthetic zone because of the minimally invasive surgical procedure, reduced treatment time, and the possibility for instantaneous tooth replacement, contributing to increased patient satisfaction [1–4].

It has been reported that implants placed in a more buccal position have a higher risk of buccal gingival recession and bone dehiscence defects [5–7]. The demand for accurate implant placement is even greater in the anterior maxilla, where optimal esthetics and long-term stability depend on correct implant positioning [8].

The utilization of computer-aided implant surgery (CAIS) can guarantee ideal implant placement, which contributes to ideal clinical outcomes and long-term stability of the soft and hard tissues of the peri-implant [9]. CAIS can be divided into static (sCAIS) and dynamic computer-aided implant surgery (dCAIS) [10]. Numerous studies have measured and validated the accuracy of implant placement using CAIS protocols [11–13]. Nevertheless, compared to implant placement in healed sites, the morphology of the sloping alveolar wall of the fresh extraction socket poses an enormous challenge to IIP [8, 14, 15]. Although previous experiments claimed that IIP with sCAIS can be performed with significantly higher accuracy than freehand, buccal shift still occurs even under fully guided surgery [12]. In addition to CAIS, implant design is a critical consideration for ideal IIP [16, 17]. The macrogeometry of an implant mainly comprises implant shape, thread design, cutting edge, and flute [18–22]. The correlation between implant macrogeometry and primary stability in IIP has been confirmed in literature [23–28]. However, the relationship between implant macrogeometry and the accuracy of guided IIP is limited [29–31].

In sCAIS, it has been revealed that the macro design of dental implants influences the accuracy of sCAIS. One study showed that tapered designs offered slightly better positional accuracy than parallel-walled macro designs, independent of the method of insertion used. Therefore, macrogeometry may also influence the accuracy of IIP in dCAIS [16]. When performing dCAIS-guided IIP, the entry point is not restrained by the guide sleeving, which might be affected more by the different slope resistance of the extraction socket, resulting in a larger deviation in the entry point. It is unknown whether the difference in macrogeometry of dental implant shape will amplify the deviation.

To the best of the authors’ knowledge, studies examining the accuracy of different macrogeometry of dental implants in dCAIS-guided IIP are currently unavailable. Therefore, this study aimed to explore and compare the accuracy of IIP between some commercially available macrogeometry with dCAIS. This experiment hypothesized that the macrogeometry of the implant affected the accuracy of IIP.

## Methods

### Study design

As the data used for the experiment were not derived from clinical cases, no ethical review was required for the model study.

Considering the lack of similar studies in the previously published literature to obtain mean values and standard deviations of implant placement accuracy under dynamic navigation for each type of implant, if α was set at 0.05, β was set at 80%, and effect size f was set at 0.4 with a 1:1:1:1 distribution ratio between four study groups, a sample size of 19 models per group was needed in a one-way ANOVA study. A one-way ANOVA study with a sample of 76 subjects divided among 4 groups achieves a power of 82% when assuming a non-central F test with a significance level of 0.05.

### Preoperative plan

**Fig. 1:**
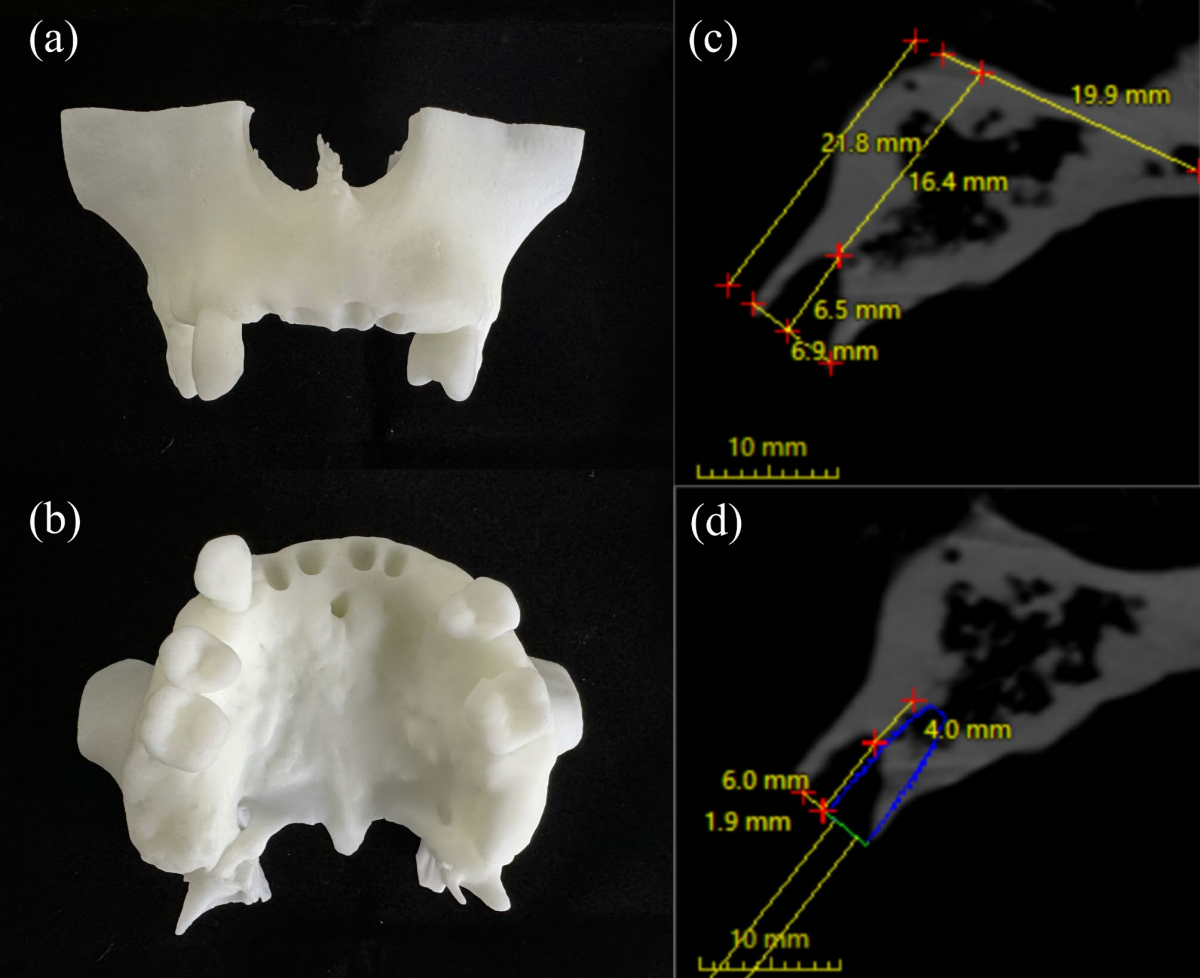
Model and digital plan (**a**) Occlusal view of the resin model; (**b**) Buccal view of the resin model; (**c**) Measurements of the extraction socket on sagittal plane of CBCT scans in the anterior maxilla; (**d**) Measurements of the planned implant relationship to the extraction socket on sagittal plane of CBCT scans in the anterior maxilla. CBCT, cone-beam computed tomography

The resin models were manufactured (WeNext Technology Co., Ltd, Shenzhen, China) according to the same STL file by the same stereolithography (SLA) 3D printer (UnionTech Co., Ltd, Shanghai, China). The maxillary central and lateral incisors were designated for IIP in this model (Fig. 1a and b). The remaining alveolar bone of the extraction socket and the planning of the implant were measured according to the measurements in a retrospective study [32]. The total height of the alveolar process was 21.8 mm, and the bone’s width at the alveolar apex level was 6.9 mm (Fig. 1c). According to Kan et al., [33] each maxillary anterior socket exhibited a sagittal position classified as Class I, which served as an indication for IIP. The total implant length was 10 mm. The implant anchored in the peri-apical bone was 4 mm, and the implant length protruding in the socket was 6 mm. The gap between the implant and the bone buccal plate was 2 mm (Fig. 1d).

### Preoperative preparation

The model experiment was conducted by a surgeon (J.C.) experienced in dCAIS. Implant osteotomies and placement were guided by an active dynamic navigation system (Yizhimei, DCARER, Suzhou, China).

**Fig. 2:**
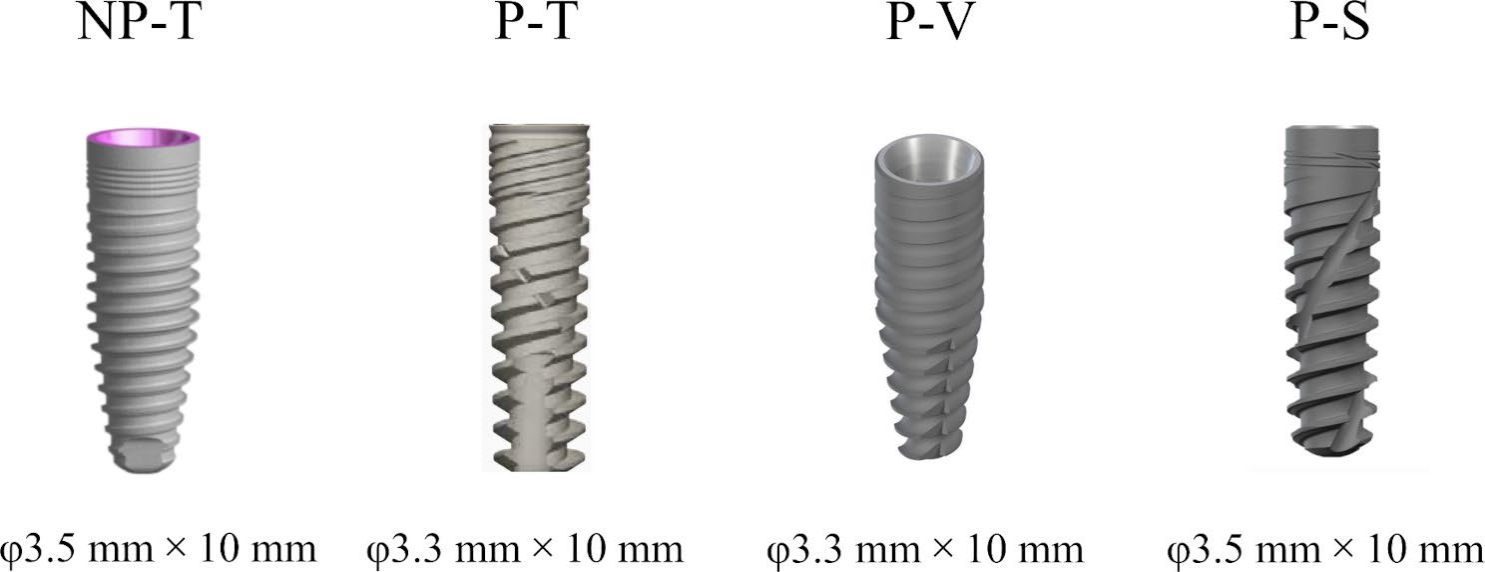
Four implant system groups with different macrogeometry: non-progressive and trapezoidal (NP-T), progressive and trapezoidal (P-T), progressive and V-shaped (P-V), progressive and spiral (P-S)

The Shore hardness of the R4600 photosensitive resin (WeNext Technology Co., Ltd, Shenzhen, China) utilized to print the models was 79, with a printing accuracy of 200 microns. The hardness of the material was utilized to simulate the dense cancellous bone of the maxilla. Nobel Replace CC (φ3.5 mm × 10 mm; Nobel Biocare, Gothenburg, Sweden), Cortex Dynamix (φ3.3 mm × 10 mm; Cortex Dental Implants Industries Ltd., Israel), BLT (φ3.3 mm × 10 mm; Straumann AG, Basel, Switzerland), BLX (φ3.5 mm × 10 mm; Straumann AG, Basel, Switzerland) were randomly assigned to four immediate sites in each model. According to the manufacturers, the Cortex Dynamix, BLT, and BLX designs, particularly their cutting elements and flutes, exhibit superior self-tapping characteristics, making them capable of achieving stable implant placement during IIP. However, those kinds of implants were designed with different thread features and cutting elements. Consequently, this study utilized Cortex Dynamix, BLT, and BLX implants designed for IIP, along with a group comprising implants without cutting elements and flutes (Nobel Replace CC) to explore the effect of implant designs on dCAIS guided IIP accuracy. Specific characteristics of the implants are illustrated in Fig. 2; Table 1. According to the thread design of the implants, they are named in the order of group NP-T (non-progressive and trapezoidal), P-T (progressive and trapezoidal), P-V (progressive and V-shaped), and P-S (progressive and spiral). A total of 19 models were utilized in this experiment. However, there is a minor deviation in the diameters of these implants above.

**Table 1:** Characteristics of the implants with different macrogeometry

Group	Implant system	Diameter (mm)	Length (mm)	Implant shape	Thread design			Cutting element	Cutting flute
					Depth (mm)	Shape	Pitch (mm)		
NP-T	NobelReplace Conical Connection	3.5	10	Apically tapered	0.32	Trapezoidal; Non- progressive	0.7	-	-
P-T	Cortex DYNAMIX	3.3	10	Apically tapered	0.1–0.27	Trapezoidal; Progressive	0.8	Two apical cutting edges	Straight; in apical part
P-V	Straumann BLT	3.3	10	Apically tapered	0.16–0.43	V-shaped; Progressive	1.1	Three cutting edges	Straight; in apical part
P-S	Straumann BLX	3.5	10	Fully tapered	0.1–0.4	Spiral; Progressive	1.1	Bi-directional cutting edges	Spiral and full length dynamic chip flute

*Progressive: The thread depth of the implant becomes progressively deeper towards the apex; Trapezoidal, square, V-shaped, and spiral were used to describe the shape of the implant thread; NP-T: Non-progressive and trapezoidal; P-T: progressive and trapezoidal; P-V: progressive and V-shaped; P-S: progressive and spiral

### Immediate implant placement surgery

A U-shaped registration device was fixed onto the residual teeth with silicone rubber materials (DMG Chemisch-Pharmazeutische, Hamburg, Germany) before preoperative cone-beam computed tomography (CBCT, Planmeca ProMax, Planmeca Oy, Helsinki, Finland). All the models underwent CBCT scanning with the same parameters: 96 kV; 8 mA; voxel size of 0.2 mm; and scanning time of 12 s.

**Fig. 3:**
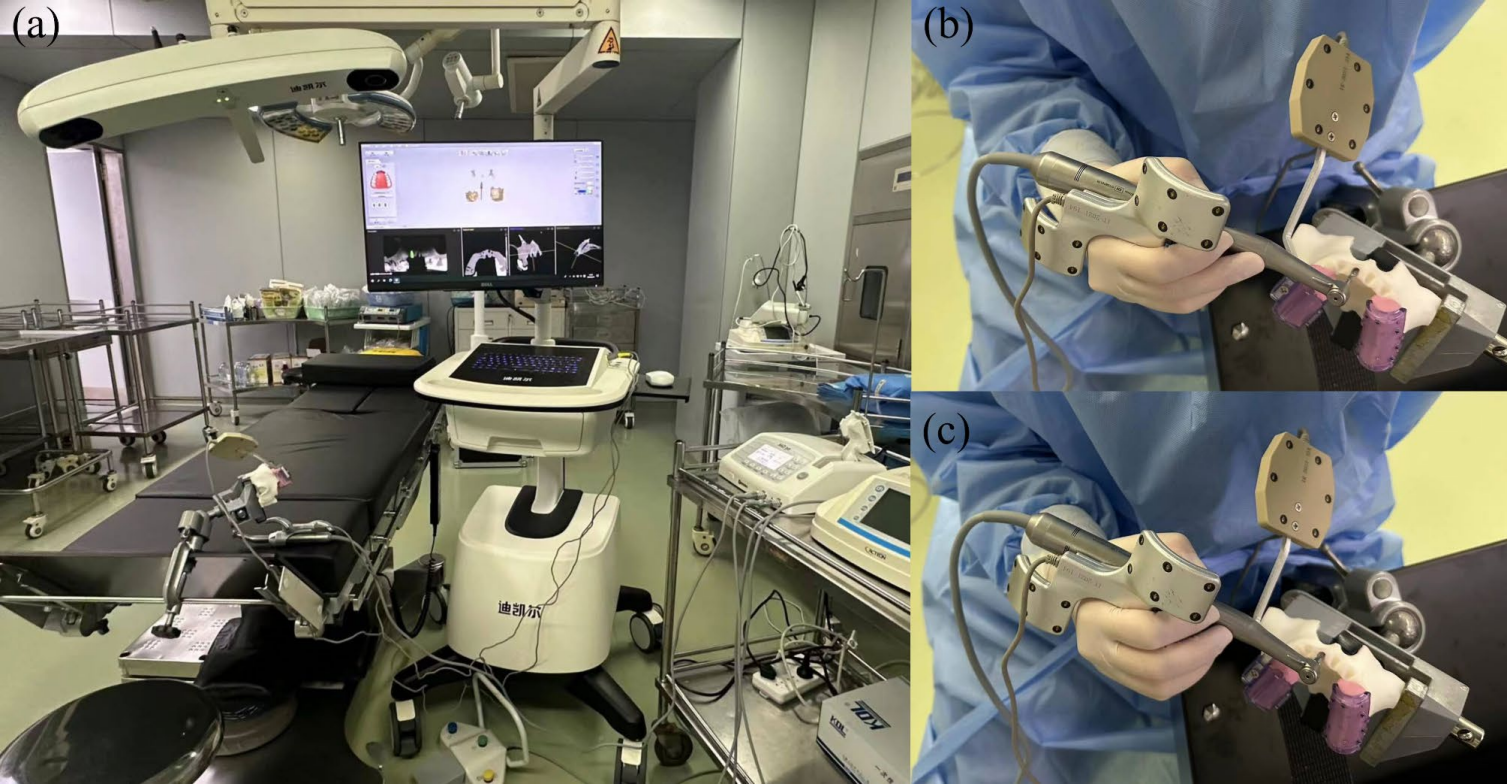
Experimental setting (**a**) Experimental environment; (**b**) Osteotomy; (**c**) Implant placement

The infrared dynamic navigation system comprises an infrared camera, a display, and a computer. Infrared cameras are equipped to detect light-emitting diodes and determine the instrument’s location and the surgical area (Fig. 3a). After fixating the model, preoperative CBCT scans were copied into software (coDiagnostiX, Dental Wings, Canada) for virtual planning of IIP. Then, the designed protocol was imported into the navigation. Based on the actual situation, the surgeon selected the appropriate handpiece and tracking plate in the dCAIS software. Subsequently, the surgeon attached short and long round burs to the handpiece separately. According to the interface instructions provided by dCAIS, the surgeon aligned the round bur with the groove located on the side of the tracking plate and rotated the handpiece, enabling dCAIS to recognize all three sides of the positioning device attached to it. The handpiece and tracking plate had to remain within the dCAIS camera’s field of view throughout the calibration procedure. Then, the tracking plate was fixed on the model with screws, connecting devices, and temporary crown materials (DMG Chemisch-Pharmazeutische, Hamburg, Germany). Afterwards, the handpiece with the short round bur (φ2.0 mm) was matched to the U-shaped tube for registration. Then, the drill was placed on the tooth cusps to check the tracking accuracy. After choosing the instrument in the software, osteotomy and implant placement were performed under navigation. The implantation site was first marked with a needle drill (φ1.6 mm). Then, a pilot drill (φ2.0 mm for NP-T and P-T/φ2.2 mm for P-V and P-S) was used to prepare the implant bed to the final preparation depth (Fig. 3b). The tapered drill widened the implant bed (φ2.8 mm for P-T, P-V, and P-S/φ3.5 mm for NP-T). In the P-V group, the coronal part of the implant bed had to be shaped with a profile drill (φ3.3 mm). At last, the implant was placed with the handpiece (Fig. 3c). The operator observed the position and direction of the tool on the screen in real-time and adjusted accordingly to perform the surgery more accurately.

### Assessment of the implant accuracy

**Fig. 4:**
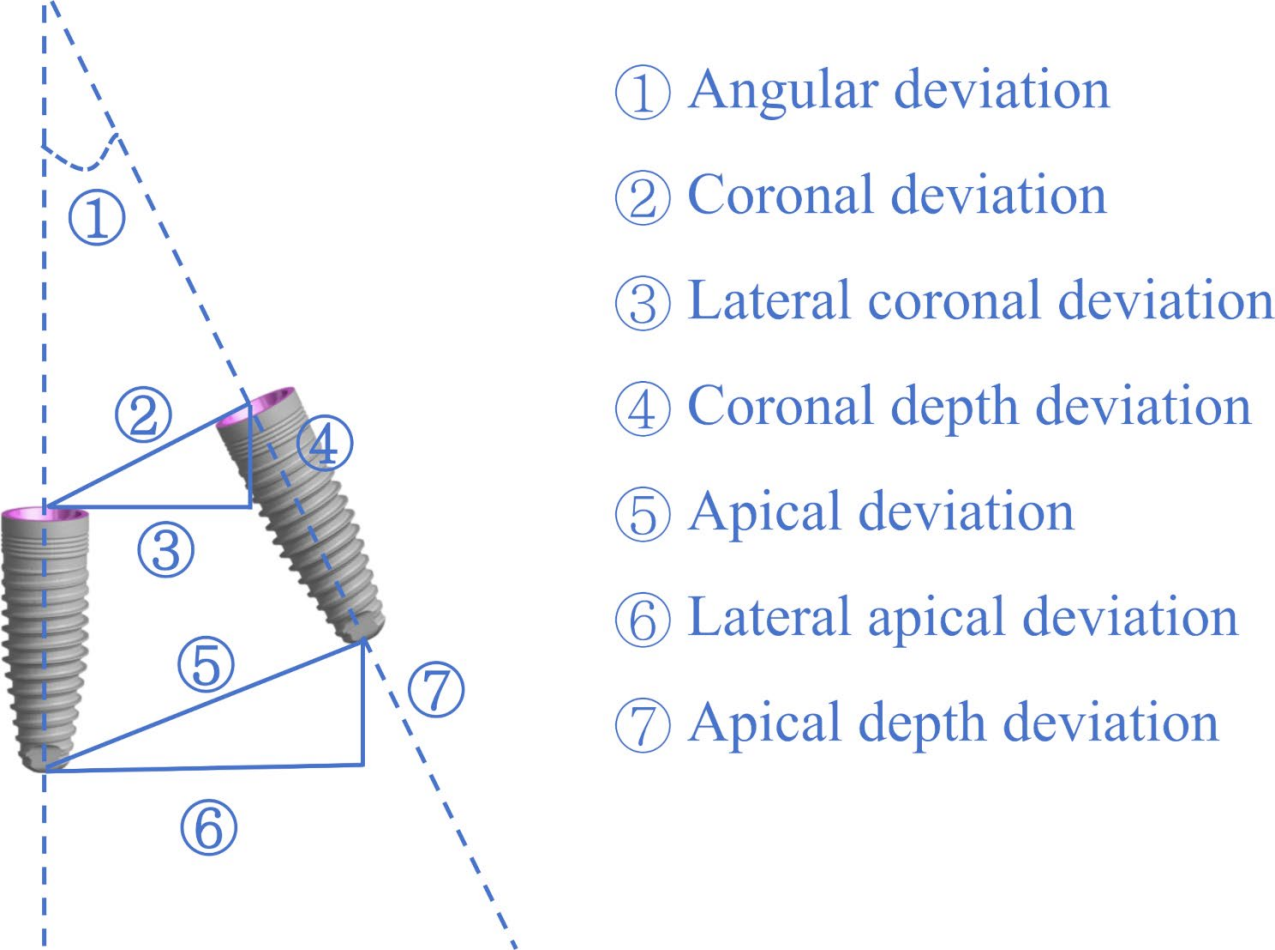
Accuracy assessment

After the surgery, postoperative CBCT was taken with the same parameters as the preoperative ones. The postoperative images were imported into the accuracy measurement software (Yizhimei, DCARER, Suzhou, China) and were fused with virtual plans based on the selected reference points. To maximize alignment accuracy, five distinct points on the U-shaped registration device were chosen, as they are the most discernible in CBCT images. Furthermore, to maintain consistency across all models, the same five points were selected and aligned in the same sequence for each measurement. The alignment accuracy was controlled within 0.25 mm. After identification of the actual implants, the coronal, coronal mesiodistal, coronal buccolingual, coronal depth, apical, apical mesiodistal, apical buccolingual, apical depth, and angular deviations between the planned and the placed implants were calculated and reported (Fig. 4). The measurements were performed by one surgeon (X.Y.) who did not participate in the execution of the surgery. The measurements were repeated after a 2-week interval.

### Statistical analysis

Agreement between two repeated measurements was assessed by limits of agreement using statistical software (MedCalc Software Ltd, Ostend, Belgium). Descriptive and comparative statistical analyses were performed using a software package (SPSS, version 27.0; SPSS Inc., Chicago, IL). The mean value of two measurements was calculated as the final results. For descriptive statistical parameters of the data, the number of observations, mean, standard deviation (SD), median, minimum (Min), and maximum (Max) are presented. The normal distribution of data was evaluated using the Shapiro-Wilk test, and Levene’s test checked the equality of variance. One-way ANOVA was adopted when the normal distribution and variances were equal. The Kruskal-Wallis test was used when the distribution was not normal. Welch’s test was performed when the distribution was normal, but the variances were not equal. Post hoc comparisons were analyzed with the Bonferroni test to see if the variances were equal; otherwise, Tamhane’s test was performed. A significant difference was defined as *p* < 0.05. The boxplots were generated using Chiplot (http://www.chiplot.online/).

## Results

**Fig. 5:**
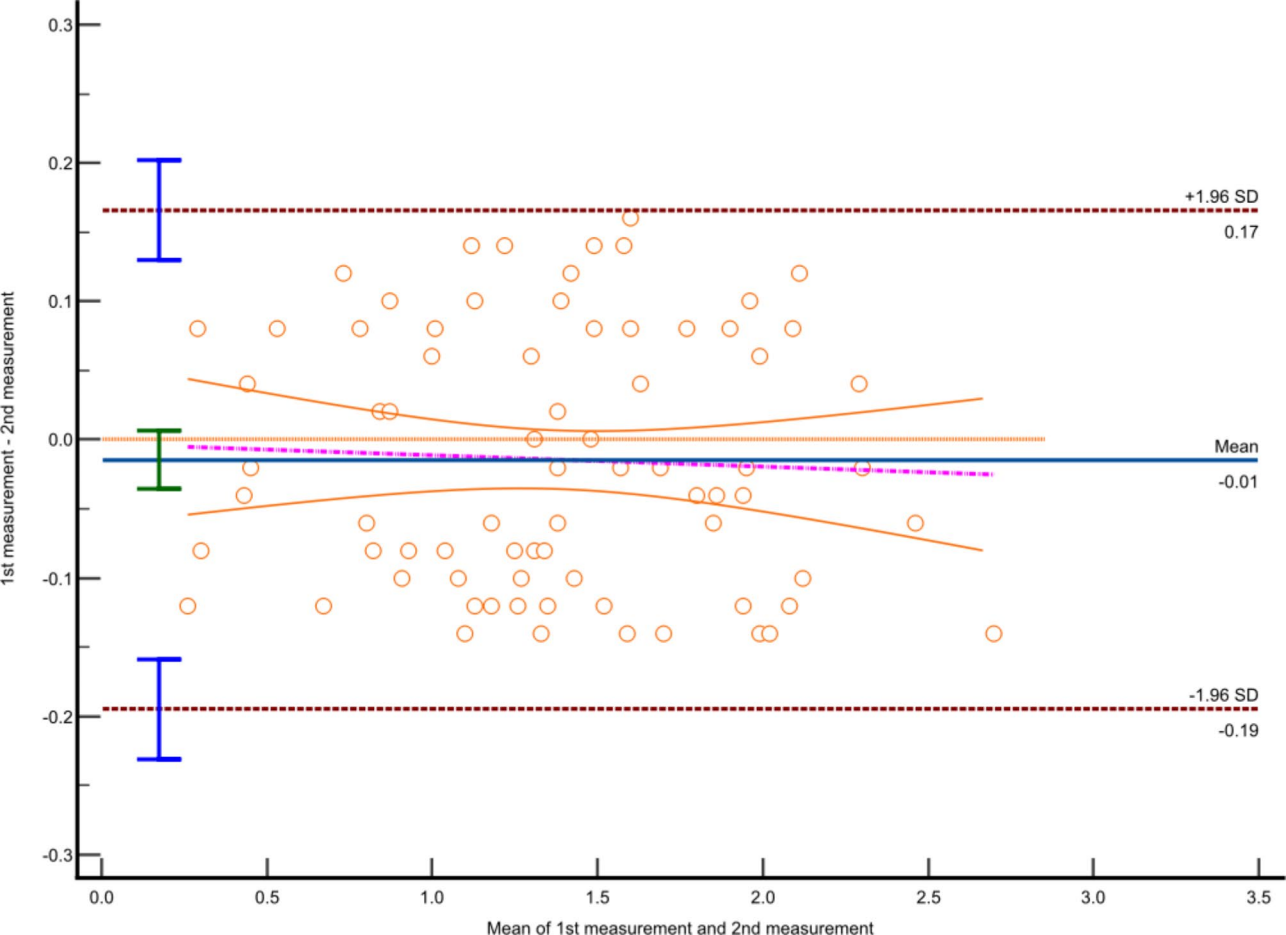
Bland-Altman plot of two repeated measurements

A total of 76 implants placed in 19 models were analyzed and illustrated (Tables 2 and 3). Bland-Altman analyses demonstrated great agreement between the two measurements (Fig. 5).

**Table 2:** Distribution and description of data in each group

	Groups	*N*	*P* value of Shapiro-Wilk test	*P* value of Levene’s test	Mean(± SD)	Median	Min	Max	*P*
Coronal deviations (mm)	NP-T	19	0.993	0.073	0.62(± 0.29)	0.58	0.05	1.27	0.035*
	P-T	19	0.863		0.46(± 0.15)	0.43	0.17	0.72	
	P-V	19	0.211		0.53(± 0.19)	0.55	0.25	1.05	
	P-S	19	0.251		0.66(± 0.22)	0.64	0.27	0.97	
Coronal buccolingual deviations (mm)	NP-T	19	0.346	0.875	-0.13(± 0.38)	-0.05	-0.68	0.81	0.001**
	P-T	19	0.809		0.08(± 0.32)	0.13	-0.58	0.65	
	P-V	19	0.072		0.06(± 0.35)	0.11	-0.97	0.56	
	P-S	19	0.171		0.34(± 0.33)	0.33	-0.21	0.81	
Coronal mesiodistal deviations (mm)	NP-T	19	0.717	0.112	-0.03(± 0.48)	-0.04	-0.97	0.81	0.218
	P-T	19	0.142		0.10(± 0.26)	0.08	-1.07	0.69	
	P-V	19	0.695		-0.05(± 0.33)	-0.04	-0.43	0.44	
	P-S	19	0.958		-0.15(± 0.40)	-0.28	-0.64	0.49	
Coronal depth deviations (mm)	NP-T	19	0.978	0.568	0.10(± 0.25)	0.11	-0.89	0.54	0.004**
	P-T	19	0.718		-0.09(± 0.29)	-0.03	-1.07	0.69	
	P-V	19	0.153		0.08(± 0.32)	0.59	-0.56	0.96	
	P-S	19	0.023*		-0.25(± 0.31)	0.79	-0.58	0.43	
Apical deviations (mm)	NP-T	19	0.085	0.244	0.66(± 0.35)	0.63	-0.33	0.67	0.047*
	P-T	19	0.419		0.50(± 0.20)	0.53	-0.87	0.66	
	P-V	19	0.001**		0.63(± 0.23)	0.59	-0.87	0.96	
	P-S	19	0.326		0.71(± 0.21)	0.79	0.17	1.68	
Apical buccolingual deviations (mm)	NP-T	19	0.334	0.933	-0.19(± 0.38)	-0.12	0.22	0.94	0.007**
	P-T	19	0.679		-0.02(± 0.36)	0.01	0.40	1.39	
	P-V	19	0.011*		0.00(± 0.42)	0.10	0.24	0.99	
	P-S	19	0.119		0.26(± 0.38)	0.43	0.17	1.68	
Apical Mesiodistal deviations (mm)	NP-T	19	0.195	0.322	-0.01(± 0.54)	0.04	-0.83	0.67	0.428
	P-T	19	0.307		0.08(± 0.31)	0.21	-0.85	0.58	
	P-V	19	0.393		-0.10(± 0.47)	-0.04	-1.31	0.51	
	P-S	19	0.931		-0.16(± 0.49)	-0.09	-0.38	0.86	
Apical depth deviations (mm)	NP-T	19	0.981	0.543	0.09(± 0.38)	0.11	-1.31	0.86	0.004**
	P-T	19	0.722		-0.09(± 0.29)	-0.04	-1.52	0.75	
	P-V	19	0.156		0.08(± 0.32)	-0.01	-0.67	0.51	
	P-S	19	0.027*		-0.25(± 0.30)	-0.29	-0.91	0.60	
Angular deviations (°)	NP-T	19	0.758	0.787	1.35(± 0.65)	1.31	-1.01	0.73	0.935
	P-T	19	0.484		1.33(± 0.45)	1.38	-1.52	0.75	
	P-V	19	0.820		1.43(± 0.58)	1.39	-0.56	0.95	
	P-S	19	0.515		1.42(± 0.50)	1.49	-0.58	0.42	

*p < 0.05; **p < 0.01; N: number of models; SD: standard deviation; Min: minimum; Max: maximum. For the Shapiro-Wilk and Levene’s tests, a *p*-value less than 0.05 would lead to the rejection of the null hypothesis, signifying that the data may not follow a normal distribution or have equal variances, respectively

**Table 3:** Post hoc comparisons

	Group(a)	Group(b)	Mean value difference(a-b)	SE	*P*
Coronal deviations	NP-T	P-T	0.16	0.07	0.172
	NP-T	P-V	0.09	0.07	1.000
	NP-T	P-S	-0.04	0.07	1.000
	P-T	P-V	-0.07	0.07	1.000
	P-T	P-S	-0.20	0.07	0.048*
	P-V	P-S	-0.12	0.07	0.548
Coronal buccolingual deviations	NP-T	P-T	-0.21	0.11	0.425
	NP-T	P-V	-0.18	0.11	0.647
	NP-T	P-S	-0.46	0.11	0.001**
	P-T	P-V	0.02	0.11	1.000
	P-T	P-S	-0.26	0.11	0.156
	P-V	P-S	-0.28	0.11	0.093
Coronal depth deviations	NP-T	P-T	0.19	7.163	0.150
	NP-T	P-V	0.01	7.163	0.933
	NP-T	P-S	0.35	7.163	0.001**
	P-T	P-V	-0.17	7.163	0.175
	P-T	P-S	0.16	7.163	0.077
	P-V	P-S	0.33	7.163	0.002**
Apical deviations	NP-T	P-T	0.16	7.163	0.497
	NP-T	P-V	0.03	7.163	1.000
	NP-T	P-S	-0.05	7.163	1.000
	P-T	P-V	-0.13	7.163	0.893
	P-T	P-S	-0.20	7.163	0.032*
	P-V	P-S	-0.07	7.163	1.000
Apical buccolingual deviations	NP-T	P-T	-0.17	7.164	1.000
	NP-T	P-V	-0.18	7.164	0.542
	NP-T	P-S	-0.45	7.164	0.003**
	P-T	P-V	-0.02	7.164	1.000
	P-T	P-S	-0.29	7.164	0.212
	P-V	P-S	-0.27	7.164	0.463
Apical depth deviations	NP-T	P-T	0.18	7.164	0.912
	NP-T	P-V	0.01	7.164	1.000
	NP-T	P-S	0.35	7.164	0.009**
	P-T	P-V	-0.17	7.164	1.000
	P-T	P-S	0.16	7.164	0.475
	P-V	P-S	0.34	7.164	0.011*

*p < 0.05; **p < 0.01; SE: standard error

In the NP-T group, the coronal, apical, and angular deviations were 0.62 ± 0.29 mm, 0.66 ± 0.35 mm, and 1.35 ± 0.65°, respectively. In the P-T group, the coronal, apical, and angular deviations were 0.46 ± 0.15 mm, 0.50 ± 0.20 mm, and 1.33 ± 0.45°, respectively. In the P-V group, the coronal, apical, and angular deviations were 0.53 ± 0.19 mm, 0.63 ± 0.23 mm, and 1.43 ± 0.58°, respectively. In the P-S group, the coronal, apical, and angular deviations were 0.66 ± 0.22 mm, 0.71 ± 0.21 mm, and 1.42 ± 0.50°, respectively. The specific deviations of the coronal and apical points in each group in the mesiodistal, buccolingual, and depth directions were presented in Table 2.

**Fig. 6:**
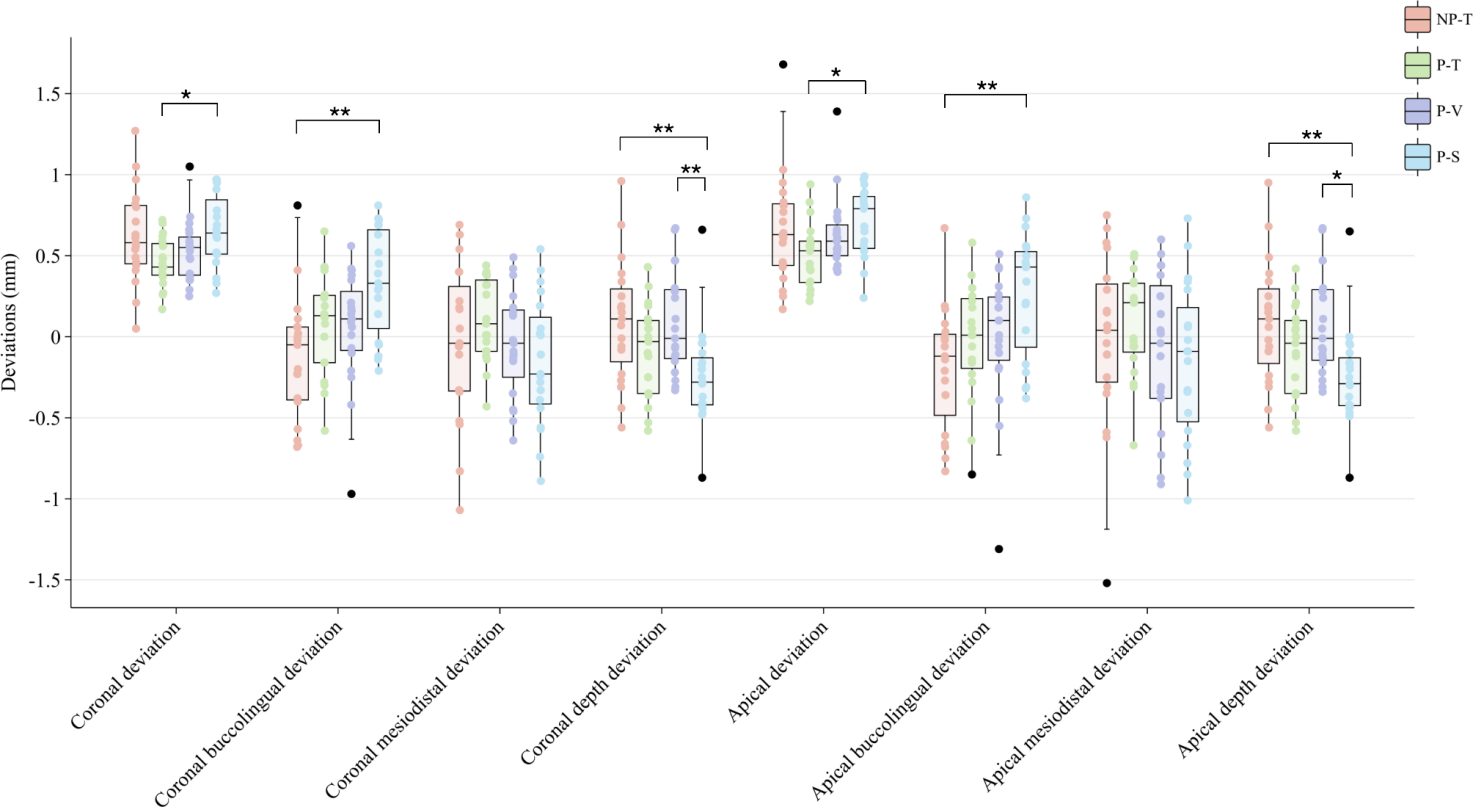
Boxplots of the distribution of the coronal, coronal mesiodistal, coronal buccolingual, coronal depth, apical, apical mesiodistal, apical buccolingual, and apical depth deviations between different groups

**Fig. 7:**
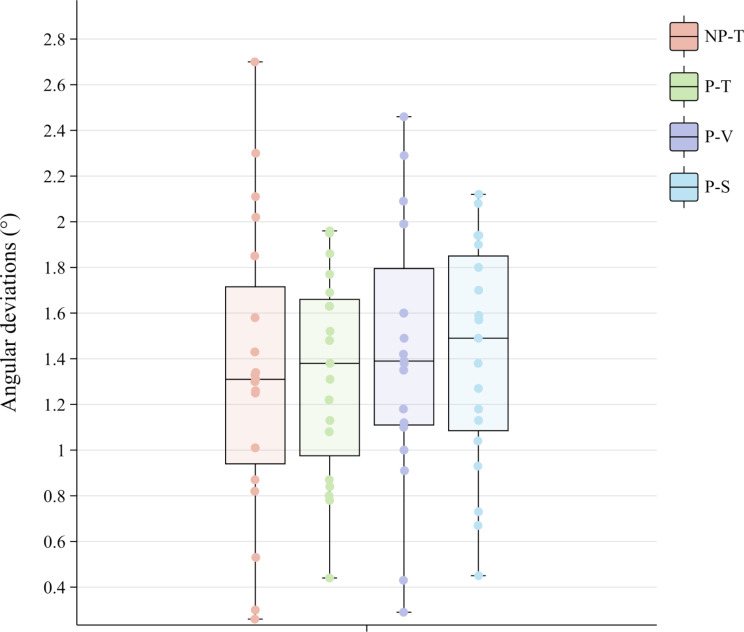
Boxplots of the distribution of the angular deviations between different groups

**Fig. 8:**
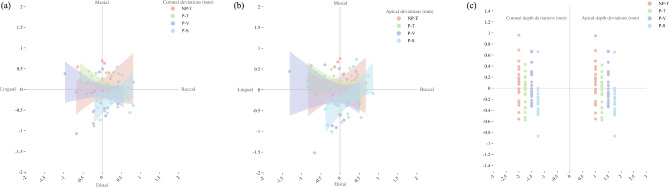
Scatterplots (**a**) Coronal buccolingual and mesiodistal distribution; (**b**) Apical buccolingual and mesiodistal distribution; (**c**) Coronal and apical depth distribution

Significant differences were detected in coronal, coronal buccolingual, coronal depth, apical, apical buccolingual, and apical depth deviations among the four groups (*p* = 0.035, *p* = 0.001, *p* = 0.004, *p* = 0.047, *p* = 0.007, *p* = 0.004, respectively) (Figs. 6 and 7) (Table 3). The P-V group demonstrated minimal coronal and apical buccolingual deviations (mean ± SD: 0.06 ± 0.35 mm and 0.00 ± 0.42 mm, respectively) for IIP with the guidance of dynamic navigation. Moreover, the P-S group demonstrated maximal coronal and apical buccolingual deviations (mean ± SD: 0.34 ± 0.33 mm and 0.26 ± 0.38 mm, respectively) (Fig. 8).

## Discussion

This study examined four commercially available implants suitable for IIP in the maxillary aesthetic zone. In the present study, significant differences were detected in coronal, coronal buccolingual, coronal depth, apical, apical buccolingual, and apical depth deviations among the four groups (*p* = 0.035, *p* = 0.001, *p* = 0.004, *p* = 0.047, *p* = 0.007, *p* = 0.004, respectively). Considering that the buccolingual deviation of the implant by the buccal force generated from the palatal bone wall is essential to IIP, the model experiment is instructive for practical clinical application.

In the maxillary aesthetic zone, IIPs are often placed 3–5 mm into the palatal bone wall of the extraction socket. The implant design of the apical third section significantly impacts the primary stability. Experiments and clinical research demonstrated that tapered implants could generate lateral pressure on the surrounding bone tissue during implant placement with higher insertion torque, which increased primary stability [26–28]. Meanwhile, implant threads are developed to facilitate implant placement, improve primary stability, and allow favourable stress distribution. Thread designs mainly include square, triangular (V-shaped), buttress, reverse buttress, and trapezoidal. Implants with deep and dense threads in the apical part of the implant are more favourable for maximizing bone-implant contact area [18, 23, 24]. Cutting edge is another important parameter, and it may benefit self-tapping properties during insertion. A dynamic experimental analysis investigated the influence of cutting flute shape (spiral, straight, and without flute) on primary stability. It was found that implants without cutting flutes exhibited higher primary stability and that a straight flute design would improve resistance to lateral load for aggressive thread implants [27].

In IIP, the limited alveolar bone apical to the implant, the unequal bone volume on the buccolingual side of the implant, and the slope of the palatal bone will all result in the buccal deviation of IIP. As previously stated, since different implant thread depths and designs can affect the implant self-tapping properties and thus the primary stability of the IIP, they are also likely to influence factors in the accuracy of dynamic navigation-guided IIP.

A previous model study concluded that the macrogeometry of dental implants might influence the accuracy of sCAIS, with tapered implants offering slightly better positional accuracy than straight implants independent of the insertion method used [29]. In that study, the same kind of implants (P-V) as utilized in the present research was employed, and the coronal, apical, and angular deviations of the P-V implant placement guided by sCAIS were reported: 0.825 ± 0.167 mm, 0.974 ± 0.14 mm, and 2.747 ± 0.306°. The coronal and apical deviations observed in the previous study were slightly more pronounced compared to the accuracy of dCAIS guided P-V implant placement in our current study (0.53 ± 0.19 mm and 0.63 ± 0.23 mm, respectively). However, the angular deviations was significantly larger in the previous study than in our current one (1.43 ± 0.58°). This discrepancy may stem from the ability of dCAIS to adjust the angle intraoperatively in real-time based on the guidance provided. A recent study investigated the effect of fixture thread depth on the positional accuracy in IIP using sCAIS in human cadavers. While the osteotomy deviations were similar between the two groups, the angular and apex deviation of the deep-threaded group (2.67 ± 2.56°; 1.04 ± 0.49 mm, respectively) were significantly larger than those of the regular-threaded group (1.61 ± 1.04°; 0.67 ± 0.41 mm, respectively) during installation (*p* < 0.05) [30]. It is noteworthy that the thread depth of the regular-threaded implant employed in this study measured 0.45 mm, closely resembling the thread depths of the implants used in our experiment. The previous study reported the coronal, apical, and angular deviations associated with sCAIS guided immediate placement of regular-threaded implants in the anterior region were 0.58 ± 0.28 mm, 0.62 ± 0.38 mm, and 1.56 ± 0.73°, respectively. The previously achieved accuracy in performing anterior IIP was comparable to the results presented in this study.

In this study, the P-V group demonstrated minimal coronal and apical buccolingual deviations (mean ± SD: 0.06 ± 0.35 mm and 0.00 ± 0.42 mm, respectively) for IIP with the guidance of dCAIS. The P-T group also exhibited ideal coronal and apical buccolingual accuracy (mean ± SD: 0.08 ± 0.32 mm and -0.02 ± 0.36 mm, respectively). Moreover, the P-S group demonstrated maximal coronal and apical buccolingual deviations (mean ± SD: 0.34 ± 0.33 mm and 0.26 ± 0.38 mm, respectively). This may be because V-shaped and trapezoidal threads have better-cutting properties than spiral threads, presenting fewer buccal deviations. The maximal difference between groups was up to 0.3 mm, which was clinically significant. This suggests that when we place implants with P-S thread shapes with the guidance of dynamic navigation, the coronal and apical buccolingual deviations need attention. The prefabricated temporary crown may not be delivered in this situation due to this deviation. In addition, the P-V and P-T implants are ideal options when multiple types of implants are available for dynamic navigation-guided IIP. The coronal and apical depth deviations of the P-S group (mean ± SD: -0.25 ± 0.31 mm and -0.25 ± 0.30 mm, respectively) were statistically different from those of the NP-T (mean ± SD: 0.10 ± 0.25 mm and 0.09 ± 0.38 mm, respectively) and P-V (mean ± SD: 0.08 ± 0.32 mm and 0.08 ± 0.32 mm, respectively) groups, probably due to the buccal deviations of the P-S implants placement, which resulted in the implant not being fully placed into the prepared socket. However, this deviation is of little significance because the surgeon can manually adjust the final depth of implant placement according to the intraoperative bone volume in actual clinical practice.

Although there were differences between groups, overall, the accuracy of dynamic navigation-guided IIP was clinically acceptable. However, robot-guided IIP may be a better choice if higher accuracy is desired, as seen in previous studies [31].

The main limitation of this study was the difficulty in replicating the clinical setting, such as heterogeneous bone density, varying anatomical characteristics, interference of saliva and blood, limited mouth opening, and possible movement of the patient. It should be recognized that IIP deviations acquired in clinical trials with the same design may be greater than the results measured in the present study. Moreover, the drilling instruments in each test group differ. Consequently, the comparison between groups may not directly reflect the comparison between different implant macrogeometry but only a general overview of the implant systems. Future studies should investigate the impact of implant macrogeometry on the accuracy of IIP in the clinical setting and with a more refined trial design.

## Conclusions

With the limitation of the in vitro study, it seems that different macrogeometry of implants might influence the accuracy of IIP in the maxillary aesthetic zone with dynamic navigation. Implants with progressive and V-shaped thread designs perform best in reducing buccolingual deviations.

## Data Availability

No datasets were generated or analysed during the current study.

## Abbreviations

CAIS: Computer-aided implant surgery
CBCT: Cone-beam computed tomography
dCAIS: Dynamic computer-aided implant surgery
IIP: Immediate implant placement
Max: Maximum
Min: Minimum
NP-T: Non-progressive and trapezoidal
P-S: Progressive and spiral
P-T: Progressive and trapezoidal
P-V: Progressive and V-shaped
sCAIS: Static computer-aided implant surgery
SD: Standard deviation
SLA: Stereolithography
